# Lignans from *Bursera fagaroides*: Chemistry, Pharmacological Effects and Molecular Mechanism. A Current Review

**DOI:** 10.3390/life11070685

**Published:** 2021-07-13

**Authors:** Mayra Antúnez-Mojica, Antonio Romero-Estrada, Israel Hurtado-Díaz, Alfonso Miranda-Molina, Laura Alvarez

**Affiliations:** 1 CONACYT-Centro de Investigaciones Químicas-IICBA, Universidad Autónoma del Estado de Morelos, Cuernavaca 62209, Morelos, Mexico; myam@uaem.mx; 2 Departamento de Madera, Celulosa y Papel, Centro Universitario de Ciencias Exactas e Ingenierías, Universidad de Guadalajara, Km 15.5 Carretera Guadalajara-Nogales, Col. Las Agujas, Zapopan 45100, Jalisco, Mexico; are@uaem.mx (A.R.-E.); ihurtado@uaem.mx (I.H.-D.); 3 LANEM-Centro de Investigaciones Químicas IICBA, Universidad Autónoma del Estado de Morelos, Avenida Universidad 1001, Cuernavaca 62209, Morelos, Mexico; alfonso_itz@tec.mx; 4 Centro de Investigaciones Químicas IICBA, Universidad Autónoma del Estado de Morelos, Avenida Universidad 1001, Cuernavaca 62209, Morelos, Mexico

**Keywords:** *Bursera fagaroides*, lignans, tubulin, cytotoxicity

## Abstract

*Bursera fagaroides* is a medicinal tree endemic to México, it belongs to the Burseraceae family and has proven antitumor activity. Modern research, performed principally with the bark extracts, have indicated that lignans are the main active constituents of *B. fagaroides,* with a high content of aryltetralin, aryldihydronaphtalene, dibenzylbutirolactone, and dibenzylbutane-type lignans as the constituents of the active extracts. In general, lignans from *B. fagaroides* exhibited potent anti-cancer activity, although antitumor, anti-bacterial, anti-protozoal, anti-inflammatory, and anti-viral properties have also been described. This review covers literature-reported lignans from *B. fagaroides*, chemical structures, nomenclature, chromatographic techniques of isolation, characterization strategies, and highlights the anti-cancer molecular mechanisms of lignans. Evaluation of the anticancer function of lignans has been extensively investigated since the cytotoxic in vitro results and in vivo assays in mice and zebrafish models to the tubulin molecular recognition by NMR. Also, we discuss the future direction for studying this important plant species and its lignan metabolites.

## 1. Introduction

Cancer is well known as one of the most important causes of morbidity and mortality worldwide, its effects in both more and less economically developed countries, and its likelihood to rank as the leading cause of death in the 21st century. It is estimated that this public health problem caused 9.6 million deaths worldwide in 2018 [1,2]. Currently, there are various types of therapies for cancer treatment, but chemotherapy is one of the most widely used. However, because of the severe side effects exhibited by commercially available drugs to treat cancer, as well as the drug resistance in tumor cells, it remains a challenge for medicinal chemistry to develop novel agents and treatment strategies to attack this public health problem [3,4]. According to the Food and Drug Administration (FDA), 246 anti-cancer drugs were approved between 1940 and 2014 and around 38% of them are natural products (or derived from them) [5]. In this context, one of the most important sources of anti-cancer secondary metabolites is the *Bursera* genus because it has been reported that these plants are effective against different types of cancer [6]. The genus *Bursera* Jacq. ex L. (Burseraceae) consists of about 105 shrubs and trees with a geographical distribution extending from the Southern U.S. to Peru and the Caribbean. In Mexico, the *Bursera* species grow principally in the tropical dry forests, where about 92 species have been described and most of them (~85%) are endemic [7,8]. *Bursera* has been divided into two subgenera (subg.), or sections: *B.* subg. *Bursera* and *B.* subg. *Elaphrium* (previously known as *Bullockia*) [8]; the bark, among other traits, mainly differentiates between them. The species of section *Bursera* have colorful trunks and peeling bark, while species of *Elaphrium* have rough and non-peeling bark [9]. Several *Bursera* species are recognized because of their characteristic production of an aromatic resin (exuded) known as “copal” that provides a chemical defense against specialized herbivores [10]. Since ancient times, copal resin has been commonly used in México and Central America as incense in religious activities [11,12]. The chemical profile of the species of *Bursera* includes flavonoids [13,14], triterpenes [15,16], sesquiterpenes [16,17], diterpenes [18], and lignans [19,20]. Most of the *Bursera* species that produce lignans are widely used in México as traditional, natural medicine due to their pharmacological properties, including analgesic, anti-inflammatory, and antitumoral properties. Also, they can help treat different illnesses, such as colds, polyps, and venereal diseases [6,21]. In general, lignans from the *Bursera* genus are secondary metabolites, known for their antioxidant, apoptotic, anti-cancer, anti-inflammatory, anti-bacterial, anti-viral, anti-fungal, and anti-protozoal properties. In particular, lignans from *B. fagaroides* have been reported to have an important anti-cancer effect [6]. This review aims to summarize literature findings on the Mexican *B. fagaroides*, such as uses in medicinal folk, pharmacological effects of its extracts and chemistry, and the biological activities of its lignans. This review focuses on the biosynthesis, chemical aspects, anti-cancer effects, and molecular mechanisms of lignans from *B. fagaroides.* The information reported in this work results from a search in ScienceDirect, PubMed, and Scifinder databases.

## 2. *Bursera fagaroides*: Description, Distribution, and Uses in the Mexican Traditional Medicine

**Figure 1:**
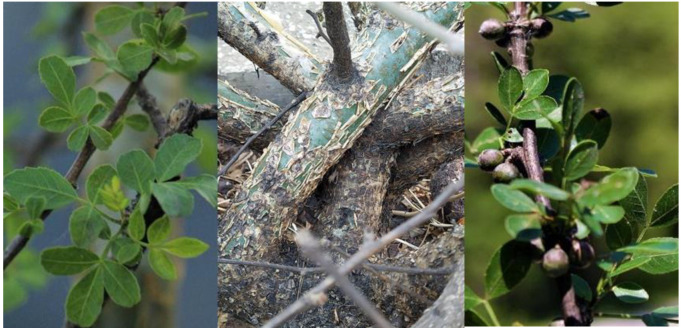
*Bursera fagaroides*, specimens from Morelos State (México). From right to left: leaves, bark, and fruits.

*Bursera fagaroides* (*B*. subg. *Bursera*) (Figure 1), also identified as *Elaphrium fagaroides*, *Amyris fagaroides*, and *Terebinthus fagaroides*, is a Mexican medicinal plant locally known as “copalillo”, “aceitillo”, “copal”, “sarzafrás” “xixote”, “cuajiote amarillo” “jiote”, “palo del diablo”, “papelillo”, and “xicote” [17,22,23,24]. It is an aromatic bush or tree of about 0.5–8 m high, distributed from the Southwestern United States of America to the Isthmus of Tehuantepec in México; it grows mainly at altitudes from 300 to 2200 m [8,17,23]. *B. fagaroides*, as traditional natural medicine, have been popularly used to treat inflammation, hits, tumors, cancer, and stomach disorders [20,22,25]. These medicinal properties have served as inspiration for various cancer research groups, as described below.

## 3. Anti-Cancer Studies of Extracts of *B. Fagaroides*

This plant species has been studied principally for its anti-cancer properties, although its antimicrobial and antigiardial effects also have been reported. An overview of the anti-cancer biological studies performed on this plant species shows that the only parts examined have been the bark and the exudate resin from the tree trunk. In vivo and in vitro studies on the extracts of these plant parts have shown important cytotoxic activities.

For instance, in 1969, Bianchi and Cole [26] found that the chloroform extract displayed a 32% reduction in the in vivo Walker carcinoma 256 tumor system (WA16). Further, the ethanol extract from the dried exudates of *B. fagaroides* showed a concentration-dependent inhibitory effect on cell proliferation against the human colon cell line HT-29, with an IC_50_ value of 0.41 ± 0.01 μg/mL at 72 h [27].

Another in vivo study by Rojas-Sepulveda [19] reported that the intraperitoneal administration of 100 mg/Kg of the hydroalcoholic extract from the bark on mice, inoculated with L5178Y lymphoma cells, increased the survival time and cured 26% (*p* < 0.001) of the treated mice. This extract also significantly inhibited the proliferation of KB (nasopharyngeal, ED_50_ = 9.6 × 10^−2^ μg/mL), PC-3 (prostate, ED_50_ = 2.5 × 10^−1^ μg/mL), HF-6 (colon, ED_50_ = 7.1 × 10^−3^ μg/mL), and MCF-7 (mama, ED_50_ = 6.6 μg/mL) tumor cell lines [19]. Later, Acevedo et al. (2015) [28] described the cytotoxicity of the *n*-hexane and chloroform extracts measured by the sulforhodamine B protein staining assay using KB, HF-6, MCF-7, and PC-3 cancer cell lines, along with a normal skin fibroblast cell line. The results indicated that both extracts displayed an important antiproliferative effect on all the studied cells, including normal cells, corroborating the results obtained previously [28].

In another in vivo study, the hydroalcoholic extract from the bark of *B. fagaroides* does not affect the number of Histone H3 phosphorylated at serine 10 (H3S10ph)-positive nuclei, with respect to the control without treatment, when measured in whole 24 h post-fertilization (hpf) zebrafish embryos; this indicated that the extract does not induce mitotic cells in the embryos [29]. This result contrasts with the strong in vivo antitumor activity against L5178Y lymphoma in mice and the authors attributed this to the poor bioavailability because of the low concentration of the active compounds present in the studied extract [19]. Further, chromatographic fractionation afforded two rich-lignans fractions that induced a high amount of cells in mitotic arrest in zebrafish embryos [29].

## 4. Lignans: Definition, Nomenclature, Biosynthesis and Pharmacological Relevance

**Figure 2:**
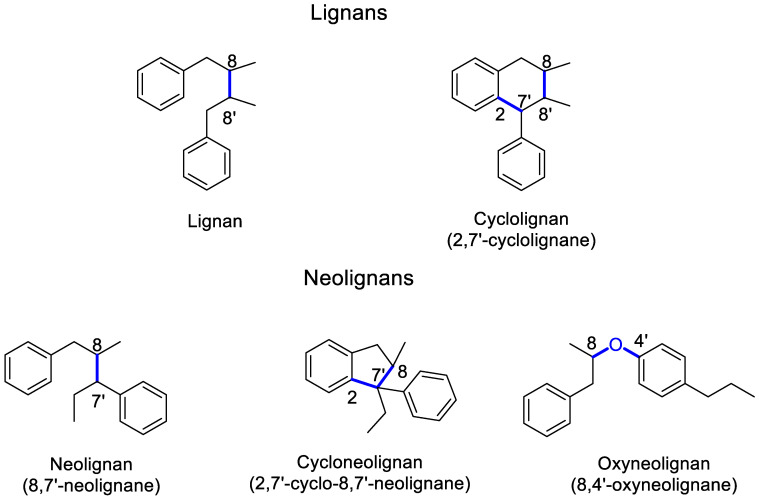
Lignans and neolignans classification, according to IUPAC recommendations [30].

Lignans and neolignans are natural products, characterized by the coupling of two phenylpropanoid units (C_6_C_3_). They can be classified into five groups according to the type and position of bond coupling the two C_6_C_3_ units and by an additional bond to form a new carbocyclic ring into lignans, neolignans, oxyneolignans, cyclolignans, and cycloneolignans. If both C_6_C_3_ units are coupled strictly in an 8–8′ position (β,β’ bond) they are named lignans (classical lignans); when coupled by other than an 8–8′ position, they give rise to neolignans. The oxyneolignans are found in the neolignans classification and are coupled through an ether bond (oxygen bridge). Also, when the lignans have an additional carbocyclic ring, they are named cyclolignans. Likewise, a neolignan with an additional carbocyclic ring gives rise to cycloneolignans [30]. Figure 2 depicts examples of five lignans and neolignans types, highlighting their differences depending on the bond/s of junction. It is also important to mention that within each group of these phenylpropanoid dimers, several subtypes can be found, such as: furofuran, furan, dibenzylbutane, dibenzylbutyrolactol, and dibenzylbutyrolactone for classical lignans. While cyclolignans can be classified as: aryltetralin, arylnaphthalene, aryldihydronaphthalene, and dibenzocyclooctadiene.

**Figure 3:**
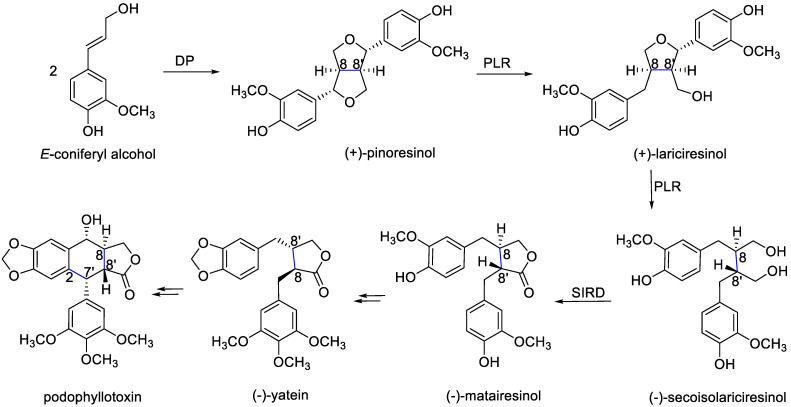
Biosynthetic pathway of lignans via shikimic acid. From the union of two monomers of coniferyl alcohol further reductions and oxidations and other modifications the different structural lignans types are created.

Regarding the lignan biosynthesis, phenylpropanoid units are generated from cinnamic acid, which in turn is formed from phenylalanine, a shikimic acid pathway metabolite (a detailed explanation of the formation of cinnamic acid derivatives can be consulted extensively at ―Shikimic Acid Pathway―) [31]. The biosynthetic pathway leading to the lignans is presented in Figure 3, where the latest enzymatic steps are still hypothetical. The enantioselective coupling of two achiral molecules of *E*-coniferyl alcohol (cinnamic acid derivative) by dirigent proteins (DP) enzymes gives rise to pinoresinol (furofuran) [32,33,34]. The (+)-pinoresinol and (-)-pinoresinol enantiomers are synthesized by *Forsythia intermedia* [32] and *Arabidopsis thaliana*, respectively [33]. Pinoresinol is reduced to lariciresinol (furan) and then to secoisolariciresinol (dibenzylbutane). Both pinoresinol and lariciresinol are substrates from PLR enzymes. Secoisolariciresinol is oxidized to matairesinol (dibenzylbutyrolactone) via the SIRD enzyme. Eventually, matairesinol is thought to be the precursor of various lignans such as yatein and podophyllotoxin (aryltetralin) [34].

Lignans have been associated with several health properties, such as protection against LDL oxidation and the inhibition of cancerous cell growth in skin, breast, prostate, colon, and lung tissues [35,36]. From a pharmacological perspective, lignans are well represented by podophyllotoxin, which generates the semisynthetic derivatives teniposide and etoposide, approved as drugs against the different types of cancer [37]. In general, these natural products possess a plethora of interesting biological properties, making them an important source of novel drug candidates and/or leading structural scaffolds exploitable in the field of medicinal chemistry.

## 5. Lignans from *B. Fagaroides*

### 5.1. Chemical Structures

**Figure 4:**
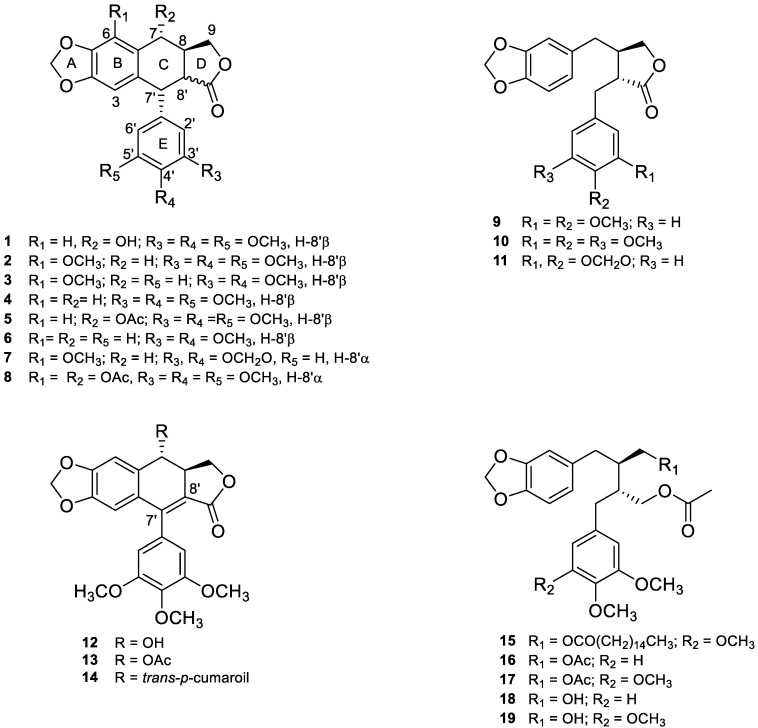
Ninteen lignans structures found in *Bursera fagaroides*. **1–8** are aryltetralin, **9–11** are dibenzylbutyrolactone, **12–14** are aryldihydronaphtalene, and **15–19** are dibenzylbutane lignans.

The chemical study of *B. fagaroides* through the years allowed the characterization of 19 lignan structures (Figure 4) named: podophyllotoxin (**1**) [19,27], β-peltatin-A-methylether (**2**) [19,26], 5′ desmethoxy-β-peltatin-A-methylether (**3**) [19,26,38], desoxypodophyllotoxin (**4**), acetyl podophyllotoxin (**5**) [19,27], morelensin (**6**) [19,27,39], burseranin (**7**) [19], acetylpicropodophyllotoxin (**8**) [40], desmethoxy-yatein (**9**), yatein (**10**) [19,40], hinokinin (**11**) [40], 7′,8′-dehydropodophyllotoxin (**12**), 7′,8′-dehydroacethyl podophyllotoxin (**13**), 7′,8′-dehydro *trans*-*p*-cumaroylpodophyllotoxin (**14**) [20], 9-acetyl-9′-pentadecanoil-dihydroclusin (**15**), 2,3-demethoxy-secoisolintetralin diacetate (**16**), dihydroclusin diacetate (**17**), 2,3-demethoxy-secoisolintetralin monoacetate (**18**) dihydroclusin mono acetate (**19**) [25]. Eight of these are aryltetralin (**1–8**), three are dibenzylbutyrolactone (**9–11**), three are aryldihydronaphtalene (**12–14**), and five are dibenzylbutane lignans (**15–19**).

Structurally nine lignans with *trans* lactone (**1–6**, **9–11**) and two with a *cis* lactone (**7** and **8**) have been found in this species. In contrast, **15–19** do not have a lactone group. All lignans have a methylenedioxy substituent in the A ring. Moreover, except for seven and eleven, lignans have at least two OMe groups in 3′,4′ position (E ring). Seven and eleven have an additional methylenedioxy substituent in the E benzene ring.

Another important chemical characteristic is the substitution in C-7, including hydroxyl (**1**), acetyl (**5** and **8**), and *trans-p*-cumaoril (**14**) groups. Finally, the most rigid skeletons are **12–14**, due to the double bound (sp^2^ hybridization) in C-7′ and C-8′.

### 5.2. Isolation and Characterization

The lignans **1–19** have been isolated from specimens of *B. fagaroides*, collected in México, principally from the bark (Michoacán state) [19,20,26,27,38,39,41,42], two reports from Oaxaca state [25] and one from Guerrero state [42] analyzed the resin. The polarity of the used extracts was chloroform (CHCl_3_) [25,26], dichloromethane (CH_2_Cl_2_) [20,38,40,41], ethanol (CH_3_CH_2_OH) [27], CH_3_CH_2_OH 80% (previously treated with hexane) [42], and methanol (CH_3_OH) 70% [19,29,39]. In general, the purification of these extracts was carried out by bioassay-guided chromatographic methods [19]. The extracts were fractionated and the components were separated by repeated column chromatography [20], eluting with gradients [19,25] or isocratic mixtures [19,38,40] of organic solvents through preparative thin layer chromatography (TLC) [20,27,38], semi-preparative reverse phase HPLC with a diode array detection system [40], flash chromatography [25], or preparative reverse phase TLC [40], as required. The yields and purity of isolated compounds were based on the peak areas of the HPLC chromatograms.

The characterization of the pure lignans **1–19** has been achieved through a complete structure elucidation, based on analyses of their spectroscopic data: High-Resolution Fast Atom Bombardment Mass Spectrometry (HRFABMS), infrared (IR), ultraviolet (UV), and circular dichroism. In addition, an extensive Nuclear Magnetic Resonance (NMR) study using standard 1D, homonuclear (COSY, TOCSY, NOESY experiments), and heteronuclear (HETCOR, gHSQC, gHMQC, gHMBC) correlated two dimensional (2D) techniques were required for general assignments of all the ^1^H and ^13^C NMR signals [6,19,20,25,26,27,38]. Other lignans were identified, comparing their physicochemical and spectroscopic data [6,43,44,45,46,47,48,49] with those reported in the literature of known lignans [50,51,52,53,54,55,56]. In general, ^1^H NMR (CDCl_3_) spectra showed signals of an unsymmetrical 1,2,4,5-tetrasubstituted [***δ***_H6_ ~ 6.83–7.2 and ***δ***_H3_ ~ 6.47–6.52] aromatic ring, one symmetrical 1,3,4,5-tetrasubstituted [H2′ and H6′, ***δ*** 6.44–6.54] aromatic ring, one oxygenated methine [***δ***H7 ~ 4.89–6.31], one oxygenated methylene [***δ***_H9a_ ~ 4.62–4.76] and [***δ***_H9b_ 4.22–4.26], three methoxyls: ***δ***_H3′_-OMe ~ 3.92–3.85, ***δ***H4′-OMe ~ 3.70–3.92, and ***δ***_H5′_-OMe ~ 3.92–3.85 for lignans **1–5**, **8**, **10**, **12–15**, **17**, and **19**, and one methylenedioxy -O–CH_2_–O- [***δ***_H_ ~ 5.99–6.05, *J* ~ 0.9 Hz and 5.99, *J* ~ 0.8 Hz] [19,20,25,26,40]. The large coupling value (*J* ~ 14.0 Hz) between H-7 and H-8 showed a *trans* diaxial relationship between these protons [20]. In the same way, the coupling constant (*J* ~ 14.5 Hz) between H-8 and H-8′ enables assigning their trans relationships [27].

The connectivity and proton-coupling network of dihydro naphthalene-type skeletons was established using a combination of ^1^H-^1^H Correlation Spectroscopy (COSY), Total Correlation Spectroscopy (TOCSY), and Nuclear Overhauser Effect Spectroscopy (NOESY) experiments, which also confirmed the presence of the aliphatic spin systems formed by the protons H-9/H-8/H-7 spin systems of aromatic protons due to the presence of two benzene rings. Coupling constants observed for these hydrogens and those observed for the adjacent ones confirmed the assignment of each proton [19,27,40]. The carbon to which each hydrogen was attached was defined from ^1^H-^13^C Heteronuclear correlation (HETCOR) [27], Heteronuclear Single Quantum Coherence (gHSQC) [19], gHMQC [27], or Heteronuclear Multiple Bond Correlation (gHMBC) experiments [19,25,27]. Full assignments of the proton and carbon resonances were secured from gHMBC, based on long-range correlation. In this context, HMBC correlations allowed to assign the ester, acetate, and methoxy groups in ring E, the position of the hydroxyl groups, and the substituted double bonds. HMBC correlations also confirmed the dihydronaphthalene skeleton [20].

In addition, their ^13^C NMR (CDCl_3_) spectra show the occurrence of carbon resonances, ascribable to carboxyl ester groups (***δ***_C_ ~ 167.7–173.7); signals at ***δ*** ~ 171.1 and ***δ*** ~ 21.0 also confirm the presence of acetyl groups; carbons at ***δ***_C7′_ ~ 147.2–147.9 and ***δ***_C8′_ ~ 118.7 in lignans **12**, **13,** and **14** were characteristics of a tetrasubstitued double bound. Other signals also were assigned: methylenedioxy group in ring A [***δ***_C_ ~ 102.0–103.1], methoxyl groups [***δ***_C_ ~58.6 - 61.2], aliphatic methylene group [***δ***_C_ ~ 69.6–70.1], aliphatic methine groups (C7) ***δ*** ~74.2–75.2 for lignans **1–8** and **12–14**, aliphatic methine groups [***δ***_C8_ ~ 41.7–43.9], characteristic signals of carbon atoms bearing oxygen at ***δ***~ 63.95 - 64.3, aromatic carbons for ring B [***δ***_C1_ ~ 128.2–130.6, ***δ***_C2_ ~ 129.1–132.0, ***δ***_C3_ ~ 110.0–110.2, ***δ***_C4_ ~ 150–153, ***δ***_C5_ ~ 147.5–148.4, and ***δ***_C6_ ~ 104.8–105.8] and those corresponding to substituted aromatic carbons for ring E [***δ***_C1′_ ~ 129.1–131.2, ***δ***_C2′_ ~ 109.6–110.0, ***δ***_C3′_ ~ 153.0–153.7, ***δ***_C4′_ ~ 135.7–139.5, ***δ***_C5′_ ~ 153.0–154.0, and ***δ***_C6′_ ~ 109.6–110.3], respectively [19,20,25,26,27,29,40]. On the other hand, the absolute configurations of lignans **4**, **5**, **7**, **11,** and **12** were determined using vibrational circular dichroism [27], while that of lignan **1** was determined by chemical correlation with *D*-phenylalanine [44] and by X-ray diffraction analysis of 2′-bromopodophyllotoxin [45].

### 5.3. Anti-Cancer Molecular Mechanism of B. fagaroides Lignans

**Figure 5:**
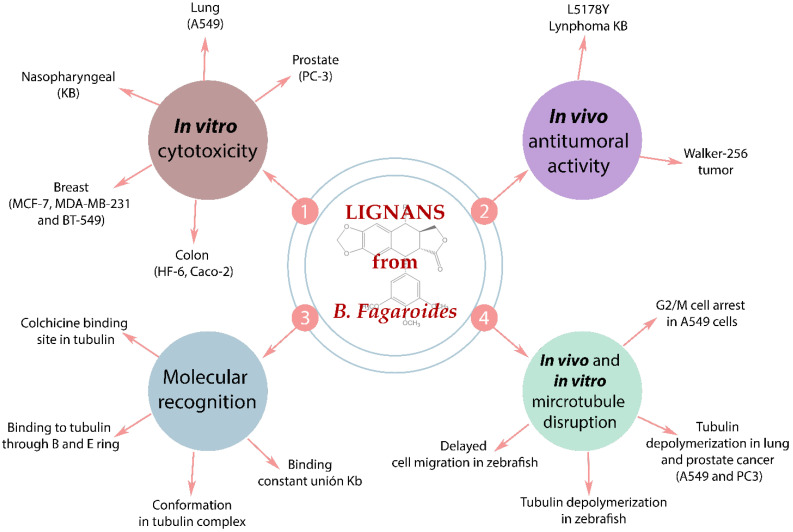
Cytotoxic, antitumoral effects, and mechanism of action of lignans isolated from *Bursera fagaroides*.

The molecular studies of lignans isolated from *B. fagaroides* are diverse and all head to anti-cancer activity. Cancer is a worldwide health problem with 9.6 million cancer deaths in 2018 [2]; due to this, lignans are eye-catching secondary metabolites from medicinal plant research implicated in cancer. Figure 5 summarizes all the assays performed for aryl tetralin and aryldihydronaphtalene lignans isolated from *B. fagaroides*, from the cytotoxic in vitro results, in vivo assays in mice and zebrafish models, and to the molecular recognition by NMR. Lignans **8**, **10**, **11**, **15–19** do not report any activity.

In vivo studies with Walker carcinoma 256 (intramuscular) were performed for **2** and **3**, they exhibited an important antitumoral activity at a level of 10% T/C at 12.5 mg/kg and 20% T/C at 100 mg/kg, respectively [26]. Also, the in vivo model using zebrafish embryos was performed to observe disruption of cell behavior; the results showed a delay cell migration in actin filaments for **1**, **2**, **5,** and **9**. Also the same compounds presented a microtubule depolymerization in the same model by α-tubulin immunofluorescence [29].

Table 1 describes the cytotoxicity (IC_50_) of 14 lignans in 9 cancer cell lines: KB, PC-3, MCF-7, MDA-MB-231, BT-549, HF-6, A549, A2780, and SiHa. According to the results, PC-3 and KB cell lines were the most sensitive with IC_50_ values between 2.29 and 4.43 × 10^−6^ µM, respectively [19,20,39,40]. β-peltatin-A-methylether (**2**) and podophyllotoxin (**1**) were the most active compounds in KB cells with an IC_50_ of 4.43 × 10^−6^ and 4.61 × 10^−6^ µM, respectively [19]. It should be highlighted that the importance of these results is due to prostate cancer occupying the first place in mortality cancer in males [57].

**Figure 6:**
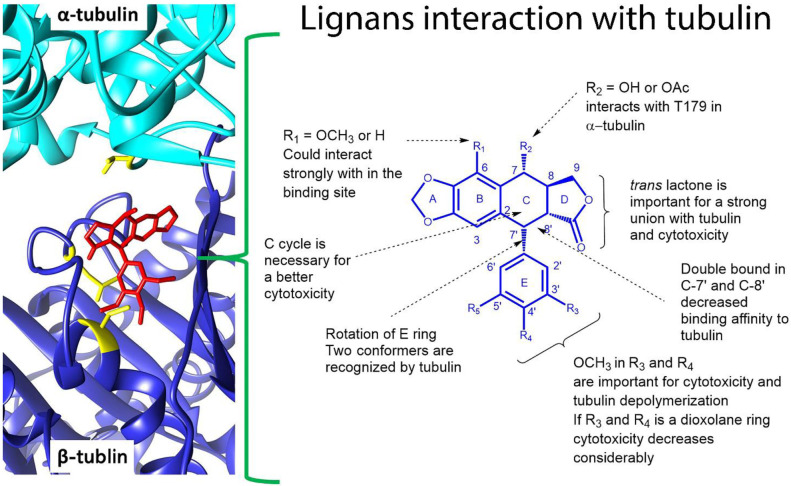
Chemical interactions of lignans in the tubulin-ligand complex (PDB 1SA0) [19,20,40,59].

It is well known that podophyllotoxin (**1**) is a cytotoxic lignan that interacts with the colchicine binding site in the α-β tubulin interphase and triggers microtubule depolymerization; this effect is related to the G2/M arrest in the cell cycle. A variety of antineoplastic drugs act with this mechanism of action such as Vinca alkaloids [58]. Based on this fact, several studies have continued to analyze podophyllotoxin-like lignans as possible anti-cancer drugs. Precisely, some cytotoxic lignans isolated from *B. fagaroides* have a podophyllotoxin type skeleton. Antunez et al., 2016 [40], determined the G2/M arrest and disrupt microtubule networks in A549 cell line for lignans **1**, **3**, **5**, **7**, and **13**. Also, mitotic nuclei (H3Ser10Ph positive) were evaluated in zebrafish models, critically; **12–14** showed a marked increase in H3S10ph positive nuclei, indicating the induction of mitotic arrest in this in vivo model [29]. A deeper description of the mechanism of action was demonstrated for cyclolignans **1**, **3**, and **5**, which bind to tubulin by the colchicine site with K_b_ values ranging from 11.75 to 185 × 10^5^ M^−1^ [40]. Figure 5 schematizes these interactions with tubulin, for instance, the double bound in C-7′-C-8′ present in **12–14** decreases the affinity to tubulin, but they still are cytotoxic with high IC_50_ values (2.42 × 10^−5^ to > 9.7 µM) [40]. Also, an NMR molecular recognition study using a STD-NMR experiment indicated that the protons in the B and E rings can interact with tubulin in the complex; further, TR-NOESY NMR established that the E ring could rotate and two rotamers can be recognized by tubulin (Figure 6) [40]. According to the previous reports [59], the enterolactone-like structure is crucial for bioactivity, specifically *trans* lactone and OMe groups in E ring are fundamental to keep the cytotoxicity, these chemical conditions were observed in the majority of the evaluated lignans. The lack of a methoxy group in 3′, 4′, and 5′ position, such as in burseranin (**7**), decreases the cytotoxicity considerably and the tubulin union is almost null.

## 6. Other Effects

Presumably, the most studied member of the lignan family is podophyllotoxin (**1**), which is used in the form of a medical cream to treat genital warts (*Condyloma acuminatum*) caused by human papillomavirus (HPV) and other venereal warts [60]. However, podophyllotoxin is also known for its severe secondary effects [61]. Despite these facts, the antiviral action of podophyllotoxin has attracted a great interest of the scientific community and its role of action was systematically studied [60]. In this regard, *B. fagaroides* lignans have not being extensively studied against HPV, but others type of lignans have been demonstrated to be HPV inhibitors [62].

It was found that on a molecular level, podophyllotoxin prevents cell division through binding to tubulin and, thus, destabilizes microtubules [29,58]. Microtubules control diverse cell functions related with the specific cell shape, motility of the cell, cell trafficking, and cell division. Thus, this action mechanism could explain the antiviral, antifungal, antibacterial, and anti-cancer activities displayed by podophyllotoxin and its congeners.

Several lignans have been found to possess these activities [43]. However, lignans isolated from *B. fagaroides* have been studied mostly for their cytotoxic and antitumor properties; only a few have been described to possess other activities [6]. For instance, podophyllotoxin (**1**), together with other aliphatic compounds, was detected as a component of the antileishmanial active extract of *B. aptera* [63]. Another study describes the in vitro effect of an ethanolic extract of *B. fagaroides* on Ornithine decarboxylase enzymes activity and on the growth of *Entamoeba histolytica* [42]. Hinokinin (**11**) was shown to have anti-inflammatory [64,65], antibacterial [66,67], antiviral [68], neuroprotective [69], and trypanosomicidal activities [70,71].

Other studies showed that another dibenzylbutirolactone lignan, yatein (**10**), can suppress herpes simplex virus type 1 (HSV-1) replication in HeLa cells [72]. Furthermore, yatein (**10**) was demonstrated to be a potent CYP3A4 inhibitor and induced herb-drug interactions in clinical situations [73]. Lignan **10** showed other important biological activities, such as anti-platelet aggregation [74].

On the other hand, considering the traditional use of *B. fagaroides* as an antidiarrheic, Gutiérrez-Gutiérrez et al. [38] demonstrated that acetylpodophyllotoxin (**5**) displayed direct antigiardial killing activity and low toxicity in Caco-2 cells. More recently, the same research group demonstrated that 5′-desmethoxy-peltatin-A-methylether (**3**), acetylpodophyllotoxin (**5**), and podophyllotoxin (**1**) affect the pattern of microtubular structures on *Giardia* trophozoites. A docking study revealed that the lignans act via binding in a hydrophobic pocket in the heterodimer interface of tubulin in *Giardia* [41].

## 7. Conclusions and Prospect

Various studies have allowed the characterizing of the cytotoxic activity of the interesting traditional Mexican medicinal plant *Bursera fagaroides,* which contains an important number of aryltetralin, aryldihydronaphtalene, dibenzylbutirolactone, and dibenzylbutane-type lignans with cytotoxic and antitumoral activity. Indeed, extracts, fractions, and pure compounds from *B. fagaroides* display important cytotoxic activity against several cancer cell lines. Also in vivo studies in mice and zebrafish embryo models demonstrated that the hydroalcoholic extract and some isolated lignans promote mitotic arrest, delay cell migration, and disrupt microtubules. Further, biochemical in vitro studies showed that this important family of compounds are potent microtubule assembly inhibitors, displaying binding to the colchicine site of tubulin. From the structural point of view, it was demonstrated that B and E rings are the major points of interaction with tubulin, the presence of the methoxyl groups at E ring, and the *trans* lactone are *sine qua non* conditions for the activity. In general, lignans obtained from *B. fagaroides* are important secondary metabolites with promising pharmacological anti-cancer effects and it could be interesting to explore them as antivirals. These compounds can act by the same mechanism of action of podophyllotoxin and can be considered in clinical trials for cancer.

## Figures and Tables

**Table 1:** Cytotoxicity of lignans in diverse carcinoma cell lines (IC_50_ in µM).

Compound	KB	PC-3	MCF-7	HF-6	A549	A2780	MDA-MB-231	BT-549	Ref
**1**	4.61 × 10^−6^	2.29	2.51 × 10^−5^	4.34 × 10^−4^	0.015	0.018	NR	NR	[19,40]
**2**	4.43 × 10^−6^	2.21	2.24 × 10^−5^	4.2 × 10^−4^	NR	NR	NR	NR	[19]
**3**	2.19 × 10^−5^	2.51 × 10^−5^	2.56 × 10^−4^ and 7.22	1.00	0.033	0.084	2.44	1.26	[19,39,40]
**4**	3.76	5.02 × 10^−3^	3.76	3.09	NR	NR	NR	NR	[19]
**5**	2.25	0.0109	>8 and 0.132	5.28	0.025	0.034	0.18	0.011	[19,39,40]
**6**	NR	NR	0.040	NR	NR	NR	0.14	0.021	[39]
**7**	7.54	5.22 × 10^−3^	9.60	7.54	8.67	12.94	NR	NR	[19,40]
**9**	1.07	4.58 × 10^−3^	1.07	1.83	NR	NR	NR	NR	[19]
**12**	0.25	2.42 × 10^−5^	>9.7	0.012	NR	NR	NR	NR	[20]
**13**	0.29	0.06	>8.8 and 0.35	0.066	NR	NR	0.16	0.06	[20,40]
**14**	3.61	2.42 × 10^−5^	>7.2	1.27	NR	NR	NR	NR	[20]

KB (nasopharyngeal), PC-3 (prostate), MCF-7, MDA-MB-231, BT-549 (breast), HF-6 (colon), A549 (lung), and A2780 (ovary). Podophyllotoxin, (**1**) β-peltatin-A-methylether (**2**), 5′ desmethoxy-β-peltatin-A-methylether (**3**), desoxypodophyllotoxin (**4**), acetyl podophyllotoxin (**5**), morelensin (**6**), burseranin (**7**), desmethoxy-yatein (**9**), 7′,8′-dehydropodophyllotoxin (**12**), 7′,8′-dehydroacethyl podophyllotoxin (**13**), 7′,8′-dehydro *trans*-*p*-cumaroylpodophyllotoxin (**14**). The IC_50_ values were taken and converted to µM from their respective reference.

## References

[1] Mbaveng A.T., Manekeng H.T., Nguenang G.S., Dzotam J.K., Kuete V., Effertha E. (2018). Cytotoxicity of 18 Cameroonian medicinal plants against drug sensitive and multi-factorial drug resistant cancer cells. J. Ethnopharmacol..

[2] Bray F., Ferlay J., Soerjomataram I., Siegel R.L., Torre L.A., Jemal A. (2018). Global Cancer Statistics 2018: GLOBOCAN Estimates of Incidence and Mortality Worldwide for 36 Cancers in 185 Countries. CA Cancer J. Clin..

[3] Roodhart J.M.L., Daenen L.G.M., Stigter E.C.A., Prins H.J., Gerrits J., Houthuijzen J.M., Gerritsen M.G., Schipper H.S., Backer M.J.G., van Amersfoort M. (2011). Mesenchymal Stem Cells Induce Resistance to Chemotherapy through the Release of Platinum-Induced Fatty Acids. Cancer Cell.

[4] Atmaca H., Çamli Ç., Sert S. (2018). Ethanol Extract of *Pinus nigra* ssp. *pallasiana* var. *şeneriana* Inhibits Human Breast Cancer Cell Viability through Induction of Apoptosis. Celal Bayar Univ. J. Sci..

[5] Newman D.J., Cragg G.M. (2016). Natural Products as Sources of New Drugs from 1981 to 2014. J. Nat. Prod..

[6] Marcotullio M.C., Curini M., Becerra J.X. (2018). An Ethnopharmacological, Phytochemical and Pharmacological Review on Lignans from *Mexican bursera* spp.. Molecules.

[7] Becerra J.X., Noge K., Olivier S., Venable L. (2012). The monophyly of *Bursera* and its impact for divergence times of *Burseraceae*. Taxon.

[8] Rzedowski J., Medina R., Calderón G. (2005). Inventario del conocimiento taxonómico, así como de la diversidad y del endemismo regionales de las especies mexicanas de *Bursera* (*Burseraceae*). Acta Botánica Mexicana.

[9] Becerra J.X. (2003). Evolution of *Mexican Bursera* (*Burseraceae*) inferred from ITS, ETS, and 5S nuclear ribosomal DNA sequences. Mol. Phylogenetics Evol..

[10] Becerra J.X., Venable D.L., Evans P.H., Bowers W.S. (2001). Interactions between Chemical and Mechanical Defenses in the Plant *Genus Bursera* and Their Implications for Herbivores. Am. Zool..

[11] Case R.J., Tucker A.O., Maciarello M.J., Wheeler K.A. (2003). Chemistry and Ethnobotany of Commercial Incense Copals, Copal Blanco, Copal Oro, and Copal Negro of North America. Econ. Bot..

[12] Linares E., Bye R. (2008). El copal en México. Biodiversitas.

[13] Nakanishi T., Inatomi Y., Satomi A., Yamada T., Fukatsu H., Murata H., Inada A., Matsuura N., Ubukata M., Murata J. (2003). New luteolin 3-O-acylated rhamnosides from leaves of *Bursera graveolens*. Heterocycles.

[14] Souza M.P., Machado M.I.L., Braz-Filho R. (1989). Six flavonoids from *Bursera leptophloeos*. Phytochemistry.

[15] Romero-Estrada A., Maldonado-Magaña A., González-Christen J., Marquina S., Garduño-Ramírez M.L., Rodríguez-López V., Alvarez L. (2016). Anti-inflammatory and antioxidative effects of six pentacyclic triterpenes isolated from the Mexican copal resin of *Bursera copallifera*. BMC Complement. Altern. Med..

[16] Columba-Palomares M.C., Villarreal M.L., Marquina S., Romero-Estrada A., Rodríguez-López V., Zamilpa A., Alvarez L. (2018). Antiproliferative and Anti-inflammatory Acyl Glucosyl Flavones from the Leaves of *Bursera copallifera*. J. Mex. Chem. Soc..

[17] Noge K., Becerra J.X. (2009). Germacrene D, A Common Sesquiterpene in the Genus *Bursera* (*Burseraceae*). Molecules.

[18] García-Gutiérrez H.A., Cerda-García-Rojas C.M., Hernández-Hernández J.D., Román-Marín L.U., Joseph-Nathan P. (2008). Oxygenated verticillene derivatives from *Bursera suntui*. Phytochemistry.

[19] Rojas-Sepulveda A.M., Mendieta-Serrano M., Mojica M.Y., Salas-Vidal E., Marquina S., Villarreal M.L., Puebla A.M., Delgado J.I., Alvarez L. (2012). Cytotoxic Podophyllotoxin Type-Lignans from the Steam Bark of *Bursera fagaroides* var. *fagaroides*. Molecules.

[20] Antunez-Mojica M., León A., Rojas-Sepúlveda A.M., Marquina S., Mendieta-Serrano M., Salas-Vidal E., Villareal M.L., Alvarez L. (2016). Aryldihydronaphthalene-type lignans *from Bursera fagaroides* var. *Fagaroides* and their antimitotic mechanism of action. RSC Adv..

[21] Purata S.E. (2008). Uso y Manejo de los Copales Aromáticos: Resinas y Aceites.

[22] Puebla-Pérez A.M., Huacuja-Ruiz L., Rodríguez-Orozco G., Villaseñor-García M.M., Miranda-Beltrán M., Celis A., Sandoval-Ramírez L. (1998). Cytotoxic and Antitumour Activity from *Bursera fagaroides* Ethanol Extract in Mice with L5178Y Lymphoma. Phytother. Res..

[23] Rzedowski J. (2004). Las especies de *Bursera* (Burseraceae) en la cuenca superior del río Papaloapan (México). Acta Botánica Mexicana.

[24] Castañeda-Miranda A.G., Chaparro M.A.E., Pacheco-Castro A., Chaparro M.A.E., Böhnel H.N. (2020). Magnetic biomonitoring of atmospheric dust using tree leaves of *Ficus benjamina* in Querétaro (México). Environ. Monit. Assess..

[25] Morales-Serna J.A., Cruz-Galicia E., García-Ríos E., Madrigal D., Gaviño R., Cárdenas J., Salmón M. (2013). Three new diarylbutane lignans from the resin of *Bursera fagaroides*. Nat. Prod. Res..

[26] Bianchi E., Sheth K., Cole J.R. (1969). Antitumor agents from *Bursera fagaroides* (Burseraceae) (β-peltatin-A-methylether and 5′-desmethoxy-β-peltatin-A-methylether). Tetrahedron Lett..

[27] Velázquez-Jiménez R., Torres-Valencia J.M., Cerda-García-Rojas C.M., Hernández-Hernández J.D., Román-Marín L.U., Manríquez-Torres J.J., Gómez-Hurtado M.A., Valdez-Calderón A., Motilva V., García-Mauriño S. (2011). Absolute configuration of podophyllotoxin related lignans from *Bursera fagaroides* using vibrational circular dichroism. Phytochemistry.

[28] Acevedo M., Nuñez P., Gónzalez-Maya L., Cardoso-Taketa A., Villarreal M.L. (2015). Cytotoxic and Anti-inflammatory Activities of Bursera species from Mexico. J. Clin. Toxicol..

[29] Antúnez-Mojica M., Rojas-Sepúlveda A.M., Mendieta-Serrano M.A., Leticia Gonzalez-Maya L., Marquina S., Salas-Vidal E., Alvarez L. (2019). Lignans from *Bursera fagaroides* Affect in vivo Cell Behavior by Disturbing the Tubulin Cytoskeleton in Zebrafish Embryos. Molecules.

[30] Moss G.P. (2000). Nomenclature of Lignans and Neolignans (IUPAC Recommendations). Pure Appl. Chem..

[31] Talapatra S.K., Talapatra B. (2015). Shikimic Acid Pathway. Chemistry of Plant Natural Products: Stereochemistry, Conformation, Synthesis, Biology and Medicine.

[32] Davin L.B., Wang H.W., Crowell A.L., Bedgar D.L., Martin D.M., Sarkanen S., Lewis N.G. (1997). Stereoselective bimolecular phenoxy radical coupling by an auxiliary (dirigent) protein without an active center. Science.

[33] Corbin C., Drouet S., Markulin L., Auguin D., Lainé É., Davin L.B., Cort J.R., Lewis N.G., Hano C. (2018). A genome-wide analysis of the flax (*Linum usitatissimum* L.) dirigent protein family: From gene identification and evolution to differential regulation. Plant Mol. Biol..

[34] Umezawa T. (2003). Diversity in lignan biosynthesis. Phytochem. Rev..

[35] Hirano T., Fukuoka K., Oka K., Naito T., Hosaka K., Mitsuhashi H., Matsumoto Y. (1990). Antiproliferative activity of mammalian lignan derivatives against the human breast carcinoma cell line, ZR-75-1. Cancer Investig..

[36] Kardono L.B., Tsauri S., Padmawinata K., Pezzuto J.M., Kinghorn A.D. (1990). Cytotoxic constituents of the bark of *Plumeria rubra* collected in Indonesia. J. Nat. Prod..

[37] Drugbank Podophyllotoxin. https://go.drugbank.com/drugs/DB01179.

[38] Gutierrez-Gutierrez F., Puebla-Perez A.M., Gonzalez-Pozos S., Hernandez-Hernandez J.M., Perez-Rangel A., Alvarez L.P., Tapia-Pastrana G., Castillo-Romero A. (2017). Antigiardial Activity of Podophyllotoxin-Type Lignans from *Bursera fagaroides* var. *fagaroides*. Molecules.

[39] Peña-Morán O., Villareal M.L., Álvarez L., Meneses-Acosta A., Rodríguez-López V. (2016). Cytotoxicity, Post-Treatment Recovery, and Selectivity Analysis of Naturally Occurring Podophyllotoxins from *Bursera fagaroides* var. *fagaroides* on Breast Cancer Cell Lines. Molecules.

[40] Antunez-Mojica M., Rodríguez-Salarichs J., Redondo-Horcajo M., León A., Barasoain I., Canales A., Cañada F.J., Jiménez-Barbero J., Alvarez L., Díaz J.F. (2016). Structural and Biochemical Characterization of the Interaction of Tubulin with Potent Natural Analogues of Podophyllotoxin. J. Nat. Prod..

[41] Gutiérrez-Gutiérrez F., Romo-Mancillas A., Puebla-Pérez A., Hernández-Hernández J.M., Castillo-Romero A. (2019). Identification and molecular characterization of the tubulin-podophyllotoxin-type lignans binding site on *Giardia lamblia*. Chem. Biol. Drug. Des..

[42] Rosas-Arreguín P., Arteaga-Nieto P., Reynoso-Orozco R., Villagómez-Castro J.C., Sabanero-López M., Puebla-Pérez A.M., Calvo-Méndez C. (2008). *Bursera fagaroides*, effect of an ethanolic extract on ornithine decarboxylase (ODC) activity in vitro and on the growth of *Entamoeba histolytica*. Exp. Parasitol..

[43] Zálešák F., Jean-Yves D., Bon D., Pospíšil J. (2019). Lignans and Neolignans: Plant secondary metabolites as a reservoir of biologically active substances. Pharmacol. Res..

[44] Schrecker A.W., Hartwell J.L. (1956). Components of podophyllin XX. The absolute configuration of podophyllotoxin and related lignans. J. Org. Chem..

[45] Petcher T.J., Weber H.P., Kuhn M., Von Wartburg A. (1973). Crystal structure and absolute configuration of 2′-bromopodophyllotoxin—0.5 ethyl acetate. J. Chem. Soc. Perkin Trans..

[46] Arora S.K., Bates R.B., Grady R.A. (1975). Crystal and molecular structure of beta-peltatin a methyl ether. J. Org. Chem..

[47] Bates R.B., Wood J.B. (1972). Crystal and molecular structure of 5′-demothxy-.beta.-peltatin A methyl ether. J. Org. Chem..

[48] Harmatha J., Buděšínský M., Trka A. (1982). The structure of yatein. Determination of the positions, and configurations of benzyl groups in lignans of the 2,3-dibenzylbutyrolactone type. Collect. Czech. Chem. Commun..

[49] Brewer C.F., Loike J.D., Horwitz S.B., Sternlicht H., Gensler W.J. (1979). Conformational analysis of podophyllotoxin and its congeners. Structure–activity relationship in microtubule assembly. J. Med. Chem..

[50] Desai D.C., Jacob J., Almeida A., Kshirsagar R., Manju S.L. (2014). Isolation, structural elucidation and antiinflammatory activity of astragalin, (−)hinokinin, aristolactam I and aristolochic acids (I & II) from *Aristolochia indica*. Nat. Prod. Res..

[51] Hendrawati O., Woerdenbag H.J., Michiels P.J.A., Aantjes H.G., Dam A.v., Kayser O. (2011). Identification of lignans and related compounds in Anthriscus sylvestris by LC-ESI-MS/MS and LC-SPE-NMR. Phytochemistry.

[52] Sakar M.K., Er N., Dilek E., Del Olmo E., San Feliciano A. (2002). (−)-Desoxypodophyllotoxin and diterpenoids from uniperus nana Willd. berries. Acta Pharm. Turcica.

[53] Da Silva R., Heleno V.C.G., de Albuquerque S., Bastos J.K., e Silva M.L.A., Donate P.M., da Silva G.V.J. (2004). Complete assignment of ^1^H and ^13^C NMR data for three aryltetralin lignan lactones. Magn. Reson. Chem..

[54] Nakanishi T., Inatomi Y., Murata H., Shigeta K., Iida N., Inada A., Murata J., Perez Farrera M.A., Iinuma M., Tanaka T. (2005). A new and known cytotoxic aryltetralin-type lignans from stems of *Bursera graveolens*. Chem. Pharm. Bull..

[55] Jolad S.D., Wiedhopf R.M., Cole J.R. (1977). Cytotoxic agents from *Bursera morelensis* (*Burseraceae*): Deoxypodophyllotoxin and a new lignan, 5′-desmethoxydeoxypodophyllotoxin. J. Pharm. Sci..

[56] Jutiviboonsuk A., Zhang H., Tan G.T., Ma C., Hung N.V., Cuong N.M., Bunyapraphatsara N., Soejarto D.D., Fong H.H.S. (2005). Bioactive constituents from roots of Bursera tonkinensis. Phytochemistry.

[57] (2020). Cancer Facts & Figures 2020. https://www.cancer.org/research/cancer-facts-statistics/all-cancer-facts-figures/cancer-facts-figures-2020.html.

[58] Gordaliza M., Castro M.D., Miguel del Corral J.M., Feliciano A.S. (2000). Feliciano, Antitumor properties of podophyllotoxin and related compounds. Curr. Pharm. Des..

[59] Mounina G., Zhou J.Z., Lu-Yong Z. (2012). Podophyllotoxin, a medicinal agent of plant origin: Past, present and future. Chin. J. Nat. Med..

[60] Ardalani H., Avan A., Ghayour-Mobarhan M. (2017). Podophyllotoxin: A novel potential natural anti-cancer agent. Avicenna J. Phytomed..

[61] Longstaff E., Von Krogh G. (2001). Condyloma eradication: Self-therapy with 0.15–0.5% podophyllotoxin versus 20–25% podophyllin preparations—An integrated safety assessment. Regul. Toxicol. Pharmacol..

[62] Xu X.Y., Wang D.Y., Li Y.P., Deyrup S.T., Zhang H.J. (2021). Plant-derived lignans as potential antiviral agents: A systematic review. Phytochem. Rev..

[63] Nieto-Yañez O.J., Resendiz-Albor A.A., Ruiz-Hurtado P.A., Rivera-Yañez N., Rodriguez-Canales M., Rodriguez-Sosa M., Juarez-Avelar I., Rodriguez-Lopez M.G., Canales-Martinez M.M., Rodriguez-Monroy M.A. (2017). In vivo and in vitro antileishmanial effects of methanolic extract from bark of *Bursera aptera*. Afr. J. Tradit. Complement. Altern. Med..

[64] Lee D.Y., Seo K.H., Jeong R.H., Lee S.M., Kim G.S., Noh H.J., Kim S.Y., Kim G.W., Kim J.Y., Baek N.I. (2013). Anti-inflammatory lignans from the fruits of *Acanthopanax sessiliflorus*. Molecules.

[65] Lima T.S.C., Lucarini R., Volpe A.C., de Andrade C.Q.J., Souza A.M.P., Pauletti P.M., Januário A.H., Símaro G.V., Bastos J.K., Cunha W.R. (2017). In vivo and in silico anti-inflammatory mechanism of action of the semisynthetic ()-cubebin derivatives ()-hinokinin and ()-O-benzylcubebin. Bioorg. Med. Chem. Lett..

[66] De Souza Pereira J.J., Pereira A.D.P., Jandú J.J., da Paz J.A., Crovella S., dos Santos Correia M.T., de Azevêdo Silva J. (2017). *Commiphora leptophloeos* phytochemical and antimicrobial characterization. Front. Microbiol..

[67] Silva M.L.A., Coimbra H.S., Pereira A.C., Almeida V.A., Lima T.C., Costa E.S., Vinholis A.H.C., Royo V.A., Silva R., Filho A.A.S. (2007). Evaluation of *Piper cubeba* extract, ()-cubebin and its semi-synthetic derivatives against oral pathogens. Phytoth. Res..

[68] Huang R.L., Huang Y.L., Ou J.C., Chen C.C., Hsu F.L., Chang C. (2003). Screening of 25 compounds isolated from Phyllanthus species for anti-human Hepatitis B virus in vitro. Phytother. Res..

[69] Yoon J.S., Koo K.A., Ma C.J., Sung S.H., Kim Y.C. (2008). Neuroprotective lignans from *Biota orientalis* leaves. Nat. Prod. Sci..

[70] Haribabu K., Ajitha M., Mallavadhani U.V. (2015). Quantitative estimation of (-)-hinokinin, a trypanosomicidal marker in *Piper cubeba*, and some of its commercial formulations using HPLC-PDA. J. Pharm. Anal..

[71] Esperandim V.R., da Silva Ferreira D., Rezende K.C., Cunha W.R., Saraiva J., Bastos J.K., e Silva M.L., de Albuquerque S. (2013). Evaluation of the in vivo therapeutic properties of (-)-cubebin and (-)-hinokinin against *Trypanosoma cruzi*. Experim. Parasitol..

[72] Usia T., Watabe T., Kadota S., Tezuka Y. (2005). Metabolite-cytochrome P450 complex formation by methylenedioxyphenyl lignans of *Piper cubeba*: Mechanism-based inhibition. Life Sci..

[73] Chen J.J., Chang Y.L., Teng C.M., Chen I.S. (2000). Anti-platelet aggregation alkaloids and lignans from *Hernandia nymphaeifolia*. Planta Med..

[74] Picking D., Chambers B., Barker J., Shah I., Porter R., Naughton D.P., Delgoda R. (2018). Inhibition of cytochrome P450 activities by extracts of *Hyptis verticillata* Jacq.: Assessment for potential HERB-drug interactions. Molecules.

